# Characterization of group II chaperonins from an acidothermophilic archaeon *Picrophilus torridus*

**DOI:** 10.1002/2211-5463.12090

**Published:** 2016-06-14

**Authors:** Yohei Y. Yamamoto, Kanako Tsuchida, Keiichi Noguchi, Naoki Ogawa, Hiroshi Sekiguchi, Yuji C. Sasaki, Masafumi Yohda

**Affiliations:** ^1^ Department of Biotechnology and Life ScienceTokyo University of Agriculture and TechnologyKoganeiJapan; ^2^ Research Fellow of Japan Society for the Promotion of ScienceChiyoda, TokyoJapan; ^3^ Instrumentation Analysis CenterTokyo University of Agriculture and TechnologyKoganeiJapan; ^4^ Department of Integrated Science in Physics and BiologyCollege of Humanities and SciencesNihon UniversitySetagaya‐kuJapan; ^5^ Japan Synchrotron Radiation Research InstituteSayoJapan; ^6^ Graduate School of Frontier SciencesUniversity of TokyoKashiwaJapan

**Keywords:** archaea, chaperone, chaperonin, dynamics, *Picrophilus torridus*, single molecule

## Abstract

Chaperonins are a type of molecular chaperone that assist in the folding of proteins. Group II chaperonins play an important role in the proteostasis in the cytosol of archaea and eukarya. In this study, we expressed, purified, and characterized group II chaperonins from an acidothermophilic archaeon *Picrophilus torridus*. Two genes exist for group II chaperonins, and both of the gene products assemble to form double‐ring complexes similar to other archaeal group II chaperonins. One of the *Picrophilus* chaperonins, PtoCPNα, was able to refold denatured GFP at 50 °C. As expected, PtoCPNα exhibited an ATP‐dependent conformational change that is observed by the change in fluorescence and diffracted X‐ray tracking (DXT). In contrast, PtoCPNα lost its protein folding ability at moderate temperatures, becoming unable to interact with unfolded proteins. At lower temperatures, the release rate of the captured GFP from PtoCPNα was accelerated, and the affinity of denatured protein to PtoCPNα was weakened at the lower temperatures. Unexpectedly, in the DXT experiment, the fine motions were enhanced at the lower temperatures. Taken together, the results suggest that the fine tilting motions of the apical domain might correlate with the affinity of group II chaperonins for denatured proteins.

**Abbreviations**
CPN: chaperonin
CS: citrate synthase from porcine heart
DXT: diffracted X‐ray tracking
GFP: green fluorescent protein
PtoCPN: group II CPN of *Picrophilus torridus*
PtoCPNαS260C/C290S: PtoCPNα with amino acid replacements of S260C and C290S
PtoCPNα: α type group II CPN of *Picrophilus torridus*
PtoCPNβ: β type group II CPN of *Picrophilus torridus*
SEC‐MALS: size exclusion chromatography – multi‐angle light scattering
*T*. KS1 CPN: group II CPN of *T*. KS‐1
*T*. KS‐1: hyperthermophilic archaeon *Thermococcus* sp. strain KS‐1
TEM: transition electron microscopy

Chaperonins (CPNs) are ubiquitous, double‐ring‐shaped molecular chaperones that capture unfolded proteins in their cavities and assist folding of these proteins in an ATP‐dependent manner. CPNs can be subdivided into groups I and II. Group I CPNs are present in bacteria and in the mitochondria and chloroplast of eukarya. Group II CPNs are present in the cytosol of archaea and eukarya 1, 2. The detailed reaction cycle of a group I CPN was revealed by studies on *Escherichia coli* GroEL 3. The heptameric ring‐shaped complex of a cochaperonin GroES acts as a lid for the central cavity. The group II CPN does not require a GroES‐like co‐chaperonin, and has a built‐in lid that consists of a helical protrusion in the apical domain 2, 4. The built‐in lid plays an important role in the functional cycle of the group II CPN. A protein in the non‐native state first binds to the apical domain of the group II CPN in the open state and is subsequently encapsulated in the cavity by the conformational change, which is induced by ATP‐binding and hydrolysis 5.

We have been investigating the protein folding mechanism of group II CPNs using those from the hyperthermophilic archaeon, *Thermococcus* sp. strain KS1 (*T*. KS1), because its group II CPNs (*T*. KS1 CPN) exhibit relatively high *in vitro* protein folding ability and structural stability. We also revealed the detailed mechanisms for the conformational change and protein folding of group II CPNs 6, 7, 8.

The conformational change in group II CPNs induced by ATP‐binding can be divided into two phases. First, ATP‐binding appears to fix the conformation of Group II CPNs in the open state. Next, ATP‐binding induces a further conformational change from the open conformation to the closed conformation, independent of the presence or absence of K^+^. This conformational change is biphasic, consisting of an initial rapid phase and a second later phase. Allostery may be observed in the late phase of the transition with an increase in intersubunit interactions. However, this is not the completely closed conformation. In the presence of K^+^, ATP hydrolysis triggers further conformational change to the completely closed state. During this conformational change, counterclockwise rotational motion of the ring occurs. The helical protrusion plays an important role in this transition. Protein folding is mediated during or after the rotational process. However, the conformational change from the closed to the open conformation has not been studied in detail. Our previous study showed the reverse rotational motion in the ATP hydrolysis cycle 8. Thus, we propose that ADP or Pi release triggers the clockwise rotational motion to unfasten the closed conformation. Consequently, the folded protein will be released from the cavity.

In contrast, our study was partly limited by the hyperthermophilic nature of *T*. KS1 CPN. We performed characterizations at high temperatures. *T*. KS1 CPN only functions at high temperatures, i.e., higher than 50 °C and exhibits neither folding activity nor ATP‐dependent conformational change at the room temperature. Previously, we attempted to construct *T*. KS1 CPN variants that could adapt to the low temperatures 9. However, these constructs did not satisfy the requirements. Thus, we searched a group II CPN that functions in a wide range temperature with structural stability.

*Picrophilus torridus* is an extremely acidophilic and moderate thermophilic archaeon that thrives optimally at pH 0.7 and 60 °C 10, 11. This organism has a very low intracellular pH compared with other thermoacidophilic organisms. On the basis of these interesting features, a number of studies have reported on the characteristics of its cellular enzymes and metabolism. Expectedly, the enzymes and proteome of this organism have acidophilic and thermophilic properties 12, 13, 14, 15. In contrast, not a few studies have shown that the recombinant proteins of *P. torridus* exhibit the highest activity at an intermediate but not acidic pH condition. Due to its relatively moderate thermostability, the proteins of *P. torridus* can function at the approximate room temperature 16, 17, 18, 19. Thus, we expected that the group II CPN from *P. torridus* is suitable for our purpose.

In this study, we expressed, purified, and characterized group II CPNs from *P. torridus* (PtoCPNs). One of the PtoCPNs, PtoCPNα, can refold denatured GFP at 50 °C. As expected, PtoCPNα exhibited an ATP‐dependent conformational change that can be observed by fluorescence changes and diffracted X‐ray tracking (DXT). In contrast to these results, PtoCPNα losts its protein folding ability at moderate temperatures, due to the loss of its ability to interact with unfolded proteins. As the release rate of the captured GFP from PtoCPNα was accelerated at lower temperatures, the affinity of the denatured protein to PtoCPNα was weakened at the lower temperature. DXT data suggest that the fine tilting motions of the apical domain might correlate with the affinity of PtoCPN to the denatured proteins.

## Materials and methods

### Bacterial strains, plasmids, reagents, proteins, and genome

*Escherichia coli* DH5α was used for propagation of plasmids, and *E. coli* BL21(DE3) (Invitrogen, Carlsbad, CA, USA) was used for protein expression. Genomic DNA of *P. torridus* was obtained from the National Institute of Technology and Evaluation. KOD‐Plus‐Neo DNA polymerase was used for gene amplification, and restriction endonucleases were obtained from Toyobo (Osaka, Japan) and New England Biolabs Japan (Tokyo, Japan). Citrate synthase from porcine heart (CS) was obtained from Sigma‐Aldrich Japan (Tokyo, Japan). Ammonium sulfate suspension of CS was desalted on an NAP‐5 column (GE Healthcare, Buckinghamshire, UK) before use, as previously described 20. The concentrations of PtoCPNs were determined using the Bio‐Rad protein assay (Bio‐Rad, Hercules, CA, USA), with bovine serum albumin as a standard, and they are reported as molar concentrations of hexadecamer. The site‐directed mutagenesis of PtoCPNs was performed using the QuikChange site‐directed mutagenesis kit (Agilent Technologies, Santa Clara, CA, USA). Nucleotides and other reagents were purchased from Wako Pure Chemical Industries (Osaka, Japan) and Sigma‐Aldrich Japan.

### Cloning, expression, and purification of PtoCPN variants

The gene for PtoCPNα was obtained by PCR amplification using the primers, PtoCPNα Fw: 5′‐ GGGAATTCCATATGATAACGGGTCAGACGCCTATATTAATATTAAAG GAAGGTACAGAAAGGCAGCA ‐3′ and PtoCPNα Rv: 5′‐CCGCTCGAGGCCTCGATCTCTGTAATTATCTTTGTACTCCCTGGCGTG ‐3′

The forward primer contains the *Nde*I site at the initiation codon, and the reverse primer was designed on the sequence approximately 300 bp downstream of the gene. The amplified DNA was digested by *Nde*I and *Xho*I and inserted into the *Nde*I/*Xho*I site of pET23b (Merck Millipore, Billerica, MA, USA). S260C and C290S mutations were introduced using QuikChange site‐directed mutagenesis kit with primers 5′‐ACCTTCAGATAAACGACC CATGTATGATACAGAAATTCC‐3′and 5′‐GGAATTTCTGTATCATACATGGGTCGTTTATCTGAAGGT‐3′ for S260C, and 5′‐GCAAATGTTTTATTATGCCAGAAGGGCATAGATG‐3′ and 5′‐CATCTATGCCCTTCTGGCA TAATAAAACATTTGC‐3′ for S260C, respectively.

The gene for PtoCPNβ was obtained by PCR amplification using the primer pair 5′‐CATGCCATGGTAGGTGGTCAGCCGATATTCATACTTAAAGAGGGTAC‐3′ and 5′‐CCGCTCGAGTTAGTCCTCACCTGCGCCCTCGCC‐3′. The fragments were digested and ligated into the *Nde*I/*EcoR*I site of pET23d. The mutation caused by the *Nco*I site was restored by mutagenesis using the primer pair 5′‐GGAGATATACCATGATAGGTGGTCAGCCG‐3′ and 5′‐CGGCTGACCACCTATCATGGTATATCTCC‐3′. A histidine tag was inserted between the 144th and 145th amino acids using the primer pairs 5′‐GCAGCTTGACAGCCTTGCAATACACCACCATCATCACCACAAGGCTGATGATGAAGAAACACTAAAG‐3′ and 5′‐CTTTAGTGTTTCTTCATCATCAGCCTTGTGGTGATGATGGTGGTGTATTGCAAGGCTGTCAAGCTGC‐3′. Arg267 was replaced to Cys using the primers 5′‐ACCTTCAGATAAACGACCCATGTATGATACAGAAATTCC‐3′ and 5′‐GGAATTTCTGTATCATACATGGGTCGTTTATCTGAAGGT‐3′.

Both PtoCPNα and PtoCPNβ were overexpressed in the soluble fraction of *E. coli* BL21(DE3). After the removal of most *E. coli* proteins by heat treatment, PtoCPNα was purified using anion exchange chromatography. The harvested cells of PtoCPNα were suspended in 50 mm Tris‐HCl, pH 7.5 and disrupted by sonication. NaCl was added to the suspension of disrupted cells to 500 mm. The cell extract was subjected to heat treatment at 70 °C for 30 min, and the denatured proteins were removed by centrifugation (25 000 ***g***, 30 min, 4 °C). The supernatant was dialyzed into 50 mm Tris‐HCl, pH 7.5, and then applied to SuperQ ‐Toyopearl column (Tosoh, Tokyo, Japan) equilibrated with buffer A (50 mm Tris‐HCl, pH 7.5, and 25 mm MgCl_2_, 1 mm DTT). PtoCPNα appeared in the eluted fraction and was collected and incubated with the addition of ATP and (NH_4_)_2_SO_4_ at 0.25 mm and 300 mm, respectively, at 35 °C for 5 h for oligomerization. The incubated product was concentrated by ultracentrifugation using Amicon Ultra (Membrane YM‐30, Merck Millipore). The concentrated fractions containing PtoCPNα were loaded onto a gel filtration column (HiLoad 26/60 Superdex 200 prep grade, GE Healthcare) equilibrated with buffer B (50 mm Tris‐HCl, pH 7.5, and 50 mm MgCl_2_, 1 mm DTT, 0.2 mm ATP, 300 mm (NH_4_)_2_SO_4_). Purified chaperonins were concentrated by ultrafiltration, supplemented with glycerol to 20% (v/v), and stored at −80 °C.

The PtoCPNβ was isolated by affinity chromatography followed by gel filtration chromatography. The harvested cells of PtoCPNβ were suspended in 50 mm Tris‐HCl, pH 8.5 and disrupted by sonication. The suspension of disrupted cells was centrifuged at 20 000 ***g*** for 30 min at 4 °C, and the supernatant was applied to DEAE‐Toyopearl column (Tosoh, Tokyo, Japan) equilibrated with buffer C (50 mm Tris‐HCl, pH 8.5, and 25 mm MgCl_2_, 1 mm DTT). PtoCPNβ appeared in the through fraction and was collected and loaded onto an affinity column (cOmplete His‐tag Purification Resin, Sigma‐Aldrich Japan) equilibrated with buffer D (50 mm Tris‐HCl, pH 8.5, and 25 mm MgCl_2_, 500 mm NaCl, 1 mm DTT). Proteins were eluted with buffer D containing 50 mm imidazole. Eluted proteins were concentrated, purified by gel filtration chromatography, and stored as described above.

### Size exclusion chromatography – multi‐angle light scattering

The purified PtoCPN complexes were analyzed by size exclusion chromatography – multi‐angle light scattering (SEC–MALS) on a WTC‐100S5 column (Wyatt Technology, Santa Barbara, CA, USA) equipped with a multi‐angle light scattering detector (MINI DAWN, Wyatt Technology) and a differential refractive index detector (Shodex RI‐101, Showa Denko, Tokyo, Japan) by an HPLC system, PU‐980i (JASCO) at 25 °C. A 100‐μL aliquot of sample was injected into the column and eluted with buffer (50 mm Tris‐HCl pH 7.5, 25 mm MgCl_2_, 0.2 mm ATP, 300 mm (NH_4_)SO_4_, 1 mm DTT) at 1.0 mL·min^−1^. The molecular weight and protein concentration were determined according to the instructional manual (Wyatt Technology).

### Transmission electron microscopy

An aliquot of the solution containing PtoCPN variants was applied onto specimen grids covered with a thin carbon support film, which was made hydrophilic by an ion‐spattering device (HDT‐400; JEOL, Tokyo, Japan) and then negatively stained with 1% gadolinium acetate for 30 s. The images were recorded using a slow‐scan CCD camera (Gatan retractable MultiScan camera; Gatan, Inc., Pleasanton, CA, USA) under low electron dose conditions at a magnification of 50 000× in a transmission electron microscope (JEM‐1400; JEOL) operated at 120 kV.

### Thermal aggregation measurement

The thermal aggregation of porcine heart CS was monitored by measuring light scattering at 500 nm with a spectrofluorometer (FP‐6500, JASCO, Tokyo, Japan) for 20 min at 50 °C. Native CS was diluted to a final concentration of 50 nm (as a monomer) with or without 50 nm of PtoCPN variants in TKM buffer (50 mm Tris‐HCl, pH 7.5, 25 mm MgCl_2_, 100 mm KCl). The reaction mixtures were preincubated for 5 min at 50 °C and continuously stirred throughout the measurement.

### Protein folding assay

Green fluorescent protein (5 μm) was denatured in TKM buffer containing 5 mm dithiothreitol and 0.1 m HCl at room temperature. To determine the refolding activity of PtoCPN variants in 50 °C, denatured GFP was diluted 100‐fold in the preincubated TKM folding buffer with or without PtoCPN variants (100 nm) at 50 °C for 10 min. Two minutes after the addition of denatured GFP, ATP was added to the mixture to a final concentration of 1 mm. The fluorescence of GFP at 510 nm with an excitation wavelength of 396 nm was continuously monitored for 60 min using a spectrofluorometer (FP‐6500). To compare the activity of PtoCPNα at different temperatures, HKM buffer (50 mm HEPES/KOH, pH 7.4, 25 mm MgCl_2_, 100 mm KCl) was used for the folding buffer. ATP was added 5 min after the addition of denatured GFP. The reaction mixtures were continuously stirred and maintained at a specific temperature for each measurement throughout the assays. As a control, native GFP was diluted in the folding buffer without chaperonin. The fluorescence intensity of native GFP was determined to be 100%.

### Fluorescence intensity assay

The PtoCPNα was labeled with fluorescein 5‐maleimide (Invitrogen). The fluorescence spectra was measured at 60 °C with a spectrofluorometer (FP‐6500). PtoCPNα (50 nm) in TKM buffer was preincubated with or without nucleotide (1 mm) at 60 °C. The excitation wavelength was established at 493 nm, and the emission was recorded at wavelengths ranged from 500 to 750 nm.

### Diffracted X‐ray tracking

A 50‐μm thickness polyimide film (Kapton, Du Pont‐Toray, Tokyo, Japan) coated with chromium (10 nm) and gold (25 nm) by vapor deposition was used as a substrate surface for DXT. An aliquot of mutant PtoCPN solution (0.2 mg·mL^−1^) in MOPS buffer (50 mm MOPS, 100 mm KCl, 5 mm MgCl_2_, pH 7.0) was applied to the gold substrate for 2 h at 4 °C. The PtoCPN‐modified surface was rinsed with the same buffer and reacted with gold nanocrystal solution for 1–2 h at 4 °C. The gold nanocrystal‐modified PtoCPN surface was rinsed with MOPS buffer and stored in the MOPS buffer until further use. An experimental chamber was constructed of sample substrate film with a spacer of polyimide film of 50‐μm thickness. The chamber was filled with MOPS buffer containing no ATP or 1 mm ATP for DXT measurement.

The dynamics of PtoCPNs were monitored through the trajectories of the Laue spots from the gold nanocrystals, which were labeled on the chaperonins. White X‐rays, 14.0‐16.5 keV (Undulator ID gap = 31.0 mm), from the beam line BL40XU (SPring‐8, Japan) were used to record the Laue diffraction spots from the gold nanocrystals on CPNs. The X‐ray beams on the sample were 50 μm (vertical) and 50 μm (horizontal). The gold nanocrystals was exposed to the X‐ray beams 90 times every 40 ms during 3600 ms. The time‐resolved diffraction images were monitored using an X‐ray image intensifier (V5445P, Hamamatsu photonics, Hamamatsu, Japan) and a CMOS camera (C11440‐10C, Hamamatsu photonics). The specimen‐to‐sample distance was approximately 100 mm and calibrated by diffraction from gold film. The sample temperature during DXT was controlled by hot air blowers at approximately 30 °C or 50 °C (TRIAC PID, Leister, Switzerland). Gold nanocrystals were obtained by epitaxial growth on NaCl (100) substrate and were dissolved with detergent [n‐Decyl‐β‐D‐maltoside (Dojindo laboratories, Tokyo, Japan), 50 mm MOPS, pH 7.0]. The average diameter of the gold nanocrystals was estimated to be 40 nm and confirmed by AFM images. Custom software written for IGOR Pro (Wavemetrics, Lake Oswego, OR, USA) was used to analyze the diffracted spot tracks and trajectories.

### GFP releasing assay

Green fluorescent protein (5 μm) was denatured in HKM buffer containing 5 mm dithiothreitol and 0.1 m HCl at room temperature. Denatured GFP was diluted to 50‐fold in the preincubated arresting buffer (HKM buffer with 1 mm DTT) with PtoCPNα (400 nm) at 60 °C for 5 min. Five minutes after the addition of denatured GFP, the aliquot of arresting buffer was diluted 10‐fold in the preincubated assay buffer, and the composition is the same as that used for the arresting buffer, at 30 °C or 50 °C for 5 min. The fluorescence of GFP at 510 nm with excitation at 396 nm was continuously monitored for 100 s using a spectrofluorometer (FP‐6500). The reaction mixtures were continuously stirred and maintained at a specific temperature for each measurement throughout the assays. As a control, native GFP was diluted in the folding buffer without chaperonin. The fluorescence intensity of native GFP was determined to be 100%. The releasing rates of a denatured GFP from PtoCPNα were estimated as follows. A denatured GFP was captured by PtoCPNα for 5 min of incubation at 60 °C in HKM buffer containing 1 mm DTT. Because excess chaperonins were present in the buffer and the temperature was sufficiently high, denatured GFP was presumed as efficiently arrested. The folding of denatured GFP was assumed to begin immediately after dilution to assay buffer preincubated at lower temperatures. The rate constants were calculated by fitting to the data of GFP fluorescence using the following equation:

$$A\left(t\right)=A\left(\infty \right)\times [1-\frac{1}{k_{2}-k_{1}}\times \left\{k_{2}e^{-k_{1}t}-k_{1}e^{-k_{2}t}\right\}]$$

where *A*(*t*) and *A*(∞) are the relative GFP fluorescence at time t and at the infinite time after the initiation of folding, respectively. *A*(∞) also calculated with *k*_1_ and *k*_2_ (*k*_1_, the rate constant for releasing of denatured GFP from chaperonin; *k*_2_, the rate constants for the spontaneous GFP folding) by a fit using the kaleida graph program (Synergy Software, Reading, PA, USA).

## Results

### Construction and structural characterization of PtoCPNα and PtoCPNβ homo‐oligomers

The *P. torridus* genome encodes two group II CPN genes, PTO0735 and PTO1195. Because of the homologies with the α and β subunits of group II CPNs from *Thermoplasma acidophilum* 21, 22 and *Thermococcus* sp. strain KS‐1 23, their products are designated as PtoCPNα and PtoCPNβ. The gene for PtoCPNα was amplified by PCR from the genomic DNA of *P. torridus* and cloned into pET23b. The PtoCPNα gene in the constructed expression plasmid contained a point mutation that caused a 139th amino acid replacement from Asp to Gly. This amino acid residue is located at the end of helix 5, and the sequence variation was observed among various archaeal species. Thus, this mutation appears to not cause any functional deficiency. This mutation might also be due to the sequence variation in the strains of *P. torridus*. The PtoCPNβ gene was also amplified by PCR from the genomic DNA of *P. torridus* and cloned into pET23d. To introduce *Nco*I at the initiation codon, a mutation occurred at the 2nd amino acid. It was restored to the original amino acid using site‐directed mutagenesis. Finally, a hexa‐histidine tag was inserted into the loop located between the equatorial domain and intermediate domain. The nucleic acid sequence was confirmed to be same as that of the designed gene.

Both PtoCPNα and PtoCPNβ were overexpressed in the soluble fraction of *E. coli* BL21 (DE3). Because both proteins are thermostable, most of the *E. coli* proteins could be removed by heat treatment. Following heat treatment, PtoCPNα was purified using anion exchange chromatography. However, native‐PAGE showed that the purified PtoCPNα was dissociated into monomers. The group II CPN of *Methanococcus thermolithotrophicus* was also dissociated into monomers during purification, and the complex was reconstituted by incubation with Mg^2+^, ATP, and (NH_4_)_2_SO_4_ 24. The PtoCPNα oligomer was reassembled using the same method, and unassembled monomers were removed using gel filtration chromatography. The reconstituted PtoCPNα oligomer was stable during further characterization. PtoCPNβ was expressed with the insertion of a hexa‐histidine tag into the loop located between the equatorial domain and the intermediate domain. A previous study had shown that the insertion of a tag sequence into these regions did not affect the activity of CPNs 25. PtoCPNβ was isolated using affinity chromatography followed by gel filtration chromatography.

**Figure 1:**
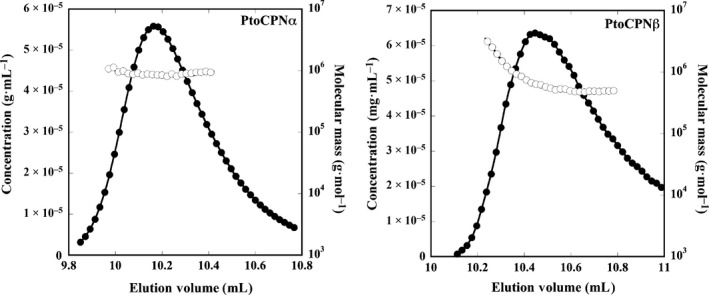
Size exclusion chromatography – multi‐angle light scattering (SEC‐MALS) of PtoCPNs. The protein concentration (closed circle) was estimated from the differential refractive index, and the molecular mass (open circle) was determined from the multi‐angle light scattering as described in Materials and methods. Left, PtoCPNα; Right, PtoCPNβ.

**Figure 2:**
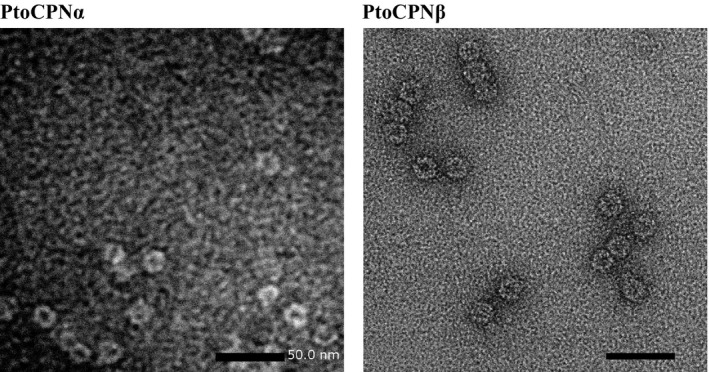
Transmission electron micrographs of PtoCPNs. Transmission electron micrographs of PtoCPNα (Left) and PtoCPNβ (Right). The black bar represents 50 nm. The details are described in the Materials and methods.

To ensure that recombinant PtoCPNs have correctly folded and assembled into a double‐ring structure, their molecular masses were determined using SEC‐MALS, Fig. 1). The molecular masses of PtoCPNα and PtoCPNβ were estimated to be 914 kDa and 865 kDa, respectively at 25 °C. In addition, their ring structures were observed using transition electron microscopy (TEM) images (Fig. 2). On the basis of these results, the double‐ring formation of these proteins was confirmed. The deduced molecular masses of the monomers of PtoCPNα and PtoCPNβ with the histidine tag were 58.8 kDa and 59.4 kDa, respectively; thus, PtoCPNα exists as a hexadecamer similar to most other group II CPNs, and PtoCPNβ appears to exist as tetradecamer. Previous studies have suggested that some archaeal group II CPNs, including those of *Sulfolobus* spp. exist as octadecamers 26. Thus, further study is required to confirm its tetradecamer structure and its physiological homo‐oligomer feature.

### Functional characterization of PtoCPNs

**Figure 3:**
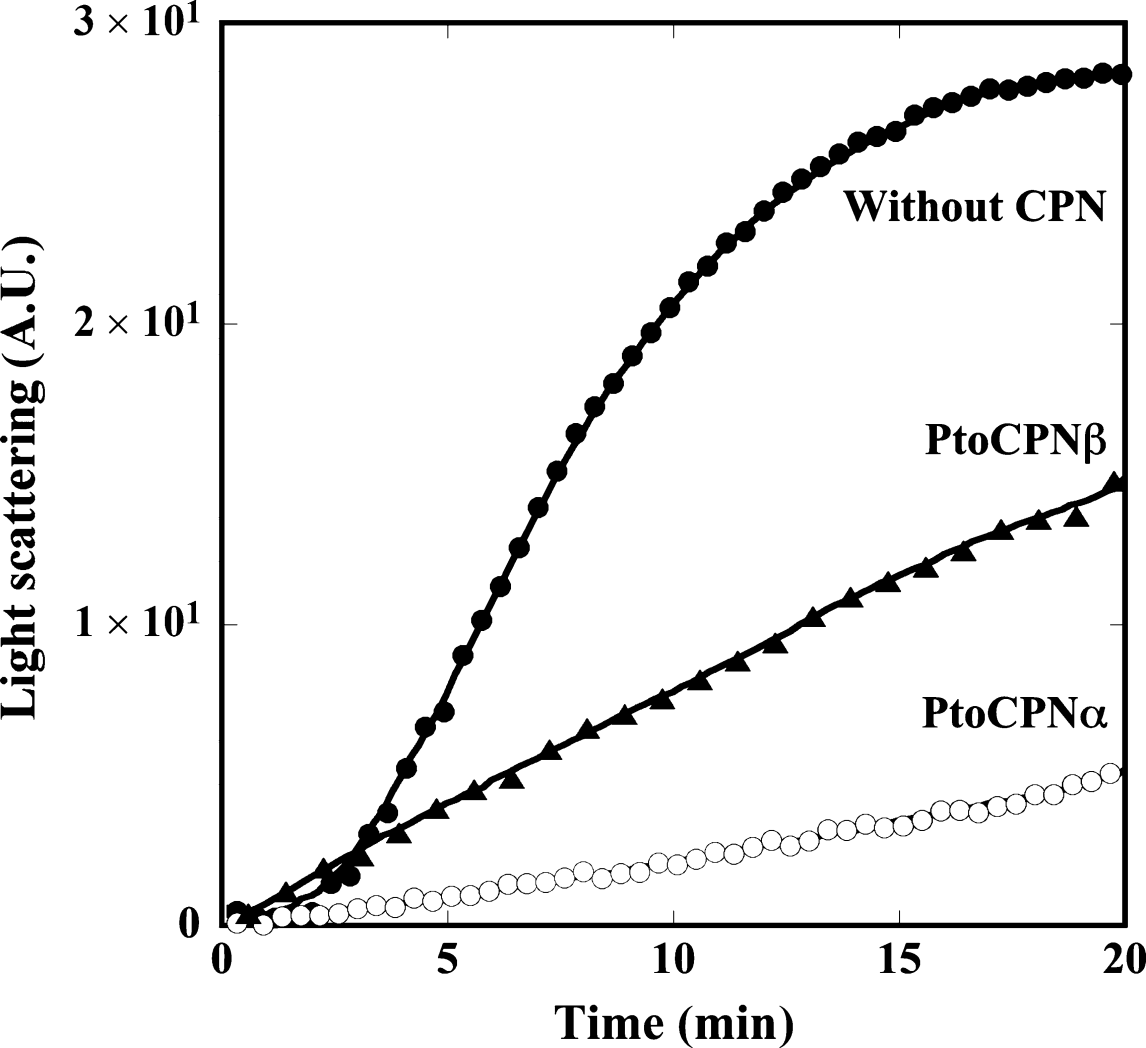
Effects of PtoCPNs on the thermal aggregation of CS. Thermal aggregation of CS at 50 °C with or without PtoCPNs was monitored using light scattering at 500 nm as described in the Materials and methods. Without CPN (closed circle), PtoCPNα (open circle) and PtoCPNβ (closed triangle).

The abilities of PtoCPNs to capture denatured proteins were evaluated using CS as a substrate. CS easily denatures and forms an aggregation at 50 °C, which is monitored as an increase in light scattering. In the presence of CPNs, the thermal aggregation of CS is repressed as CPN captures denatured CS and protects it from aggregation. Expectedly, PtoCPNs can protect CS from thermal aggregation. However, the effect of PtoCPNβ is marginal compared with that of PtoCPNα (Fig. 3). Its weak protection activity might be due to the relatively small size of the cavity compared with PtoCPNα.

**Figure 4:**
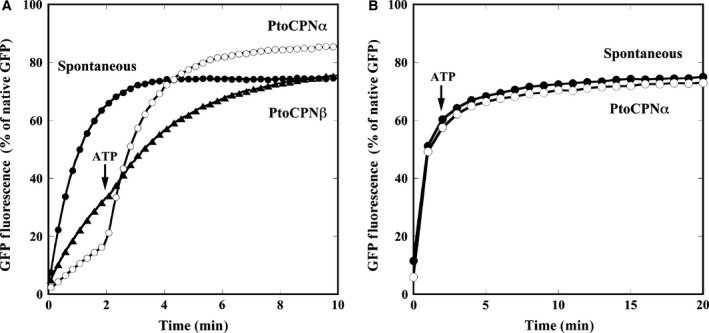
Protein refolding activity of PtoCPNs. (A) GFP refolding activity of PtoCPNα and PtoCPNβ at 60 °C. The folding mixture was incubated at 60 °C as described in the Materials and methods. The recovery of GFP fluorescence was continuously monitored at 510 nm. At 0 min, acid‐denatured GFP was diluted in the folding buffer with or without PtoCPNs. ATP was added at 2 min. Spontaneous (closed circle), PtoCPNα (open circle) and PtoCPNβ (closed triangle). (B) GFP refolding activity of PtoCPNα at 30 °C. The experiment was performed at 30 °C. Spontaneous (closed circle) and PtoCPNα (open circle).

Next, we examined the protein folding ability of PtoCPNs using acid‐denatured GFP as a substrate. Acid‐denatured GFP was diluted in the neutralization buffer in the presence or absence of PtoCPNs. The time‐dependent GFP refolding was monitored as the fluorescence recovery at 50 °C. In the absence of group II CPNs, an increase in fluorescence was observed due to the spontaneous refolding of denatured GFP. However, in the presence of active group II CPNs, the increase in fluorescence is suppressed because group II CPNs have captured denatured GFPs to prevent their spontaneous refolding. By the addition of ATP, acid‐denatured GFP refolding is enhanced by the protein refolding activity of group II CPNs. As expected, PtoCPNα arrests the spontaneous refolding of acid‐denatured GFP and enhances its refolding in an ATP‐dependent manner (Fig. 4A). In contrast, PtoCPNβ had a marginal effect on both the arrest of spontaneous refolding and ATP‐dependent fluorescence recovery (Fig. 4A). On the basis of these results, we employed PtoCPNα for further study.

Next, we examined whether PtoCPNα functions at 30 °C (Fig. 4B). Unexpectedly, PtoCPNα could not arrest the spontaneous refolding of GFP at 30 °C, and thus productive refolding was not observed. The interaction between the denatured proteins and CPNs is mainly dependent on the hydrophobic interaction 27. We speculated that the hydrophobic interaction might be weakened at lowered temperatures. The hydrophobic force is known to be strongly temperature‐dependent, as observed in some proteins that denature at low temperatures. However, the hydrophobic force peaks between 30–80 °C and becomes weaker at both lower and higher temperatures 28. Thus, it is not reasonable to propose that the interaction between CPN and denatured GFP is significantly weakened at 30 °C. In addition, GroEL can arrest the spontaneous refolding of the acid‐denatured GFP under similar conditions. Thus, we speculated that PtoCPNα might present a closed conformation at relatively cool conditions, and thus it could not interact with denatured proteins.

### Conformational change abilities of PtoCPNα

**Figure 5:**
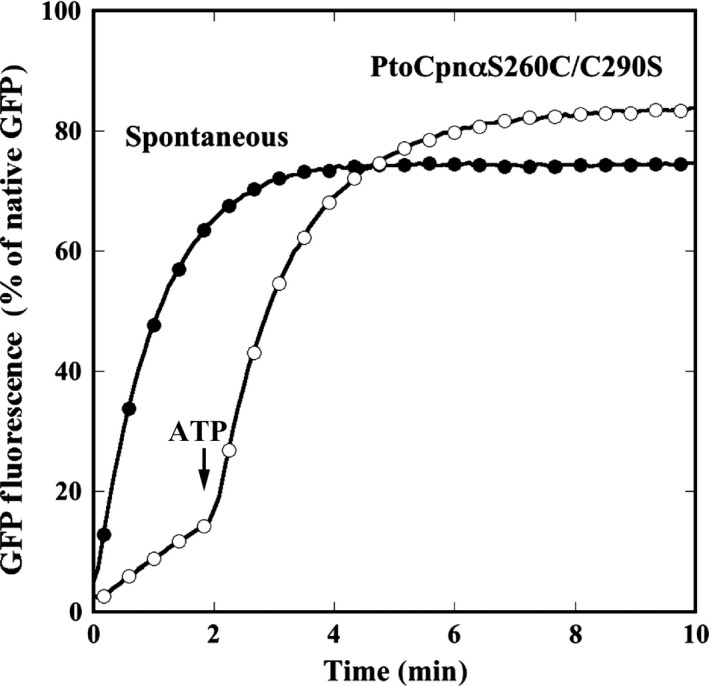
Green fluorescent protein refolding activity of PtoCPNαS260C/C290S. GFP refolding activity of PtoCPNαS260C/C290S at 60 °C. The folding mixture was incubated at 60 °C as described in the Materials and methods. ATP was added at 2 min. Spontaneous (closed circle) and PtoCPNαS260C/C290S (open circle).

**Figure 6:**
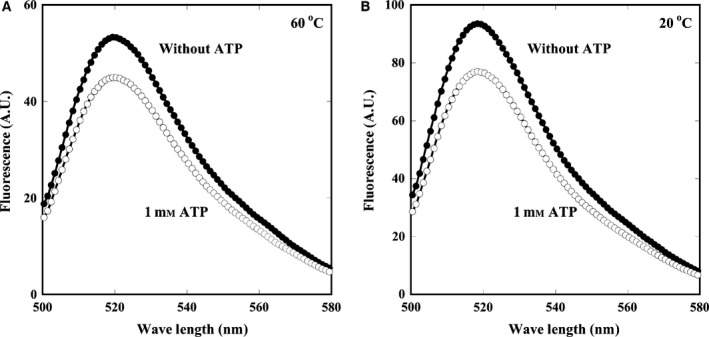
Fluorescence spectra change in fluorescein‐labeled PtoCPNα. PtoCPNαS260C/C290S was labeled with fluorescein, and their fluorescence spectra were measured by excitation at 493 nm at 60 °C (A) and 20 °C (B). Closed circle, without ATP; open circle, with 1 mm ATP. The details are described in the Materials and methods.

To examine whether PtoCPNα presents a closed conformation and has the ability to exhibit an ATP‐dependent conformational change at approximately room temperature, the ATP‐dependent conformational change in PtoCPNα was examined by the fluorescence change in the fluorophore attached to the tip of the helical protrusion. Initially, the original cysteine, Cys290, the only one cysteine in the wild‐type subunit, was converted into serine. Next, Ser260 at the tip of the helical protrusion was converted into Cys. Thus, prepared PtoCPNαS260C/C290S contains only one cysteine at the tip of the helical protrusion. PtoCPNαS260C/C290S exhibited nearly the same folding activity for the acid‐denatured GFP as wild‐type (Fig. 5). Next, the cysteine residue of PtoCPNαS260C/C290S was labeled with a fluorescein and was applied for studies on ATP‐dependent conformational change. In the open conformation, the fluorescein molecules are exposed to the hydrophilic environment. Due to the conformational change to the closed conformation, their environment converts into the hydrophobic condition, as it is surrounded by other fluorescein molecules and amino acids, resulting in a decrease in fluorescence at 520 nm 29. Fluorescein‐labeled PtoCPNα showed an ATP‐dependent fluorescence change at not only 60 °C but also 20 °C. In addition, ATP induced a decrease in the maximal fluorescence intensity of approximately 17.7% at 20 °C and 15.5% at 60 °C, respectively (Fig. 6). These results indicated that PtoCPNα alters its conformation from an open lid to a closed lid state in an ATP‐dependent manner even at lowered temperature less than 30 °C. Thus, we concluded that PtoCPNα exists as an open conformation in the absence of nucleotides, regardless of the temperature. Previously, we have shown that a *T*. KS1 CPN mutant impaired in its ATP‐hydrolyzing ability was deficient in ATP‐dependent refolding activity, although its ability to arrest GFP spontaneous refolding was partially enhanced by the addition of ATP. This mutant retained its nucleotide‐binding ability and remained in the open conformation in the presence of ATP. On the basis of these results, we showed that *T*. KS1 CPN in the absence of nucleotides is in the relatively flexible state, and ATP‐binding first fixed it in the open conformation, and a conformational change was induced into the closed form 7. It is possible that the difference in the ability to arrest GFP refolding between 30 and 50 °C might be due to the difference in the flexibility of the conformation.

### Single‐molecule analysis of the motion of PtoCPNα

**Figure 7:**
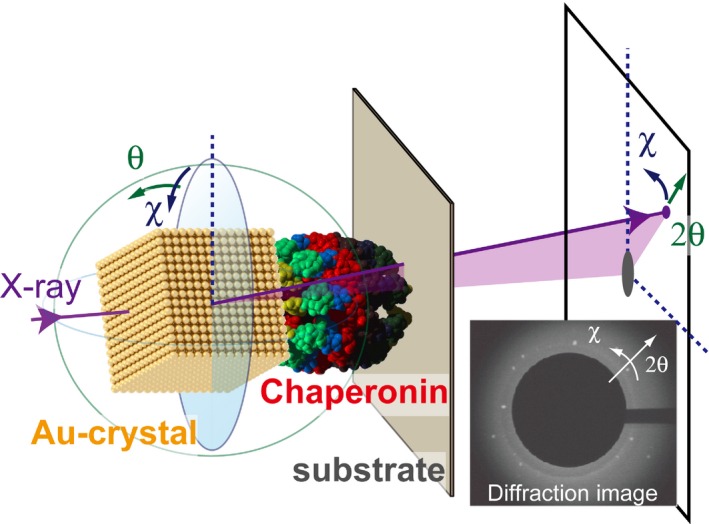
Schematic illustration of the detection of internal motions of group II chaperonins by DXT. Reproduced from 8.

**Figure 8:**
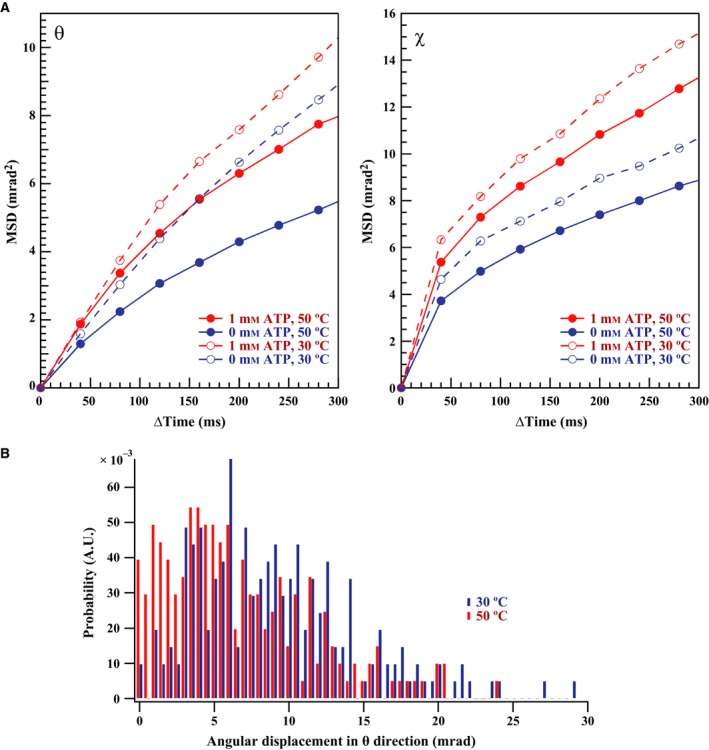
ATP‐dependent motion of PtoCPNα tracked using DXT. (A) Mean square angular displacement (MSD) in the θ and χ directions as a function of time interval in the presence of 0 mm ATP and 1 mm ATP at 30 °C and 60 °C. (B) The distribution of absolute angular displacement of PtoCPNα in the θ direction. Approximately 500 DXT trajectories were used to create the histogram at 30 °C and 60 °C. The details are described in the Materials and methods.

Previously, we analyzed the ATP‐dependent motion of *T*. KS1 CPN at the single‐molecule level using diffracted X‐ray tracking (DXT) 8 (Fig. 7). ATP hydrolysis induces rotational motion, which correlates with protein folding. In addition, DXT provides detailed information of the fine motion of *T*. KS1 CPN. Next, we analyzed the ATP‐dependent motion of PtoCPNα using DXT. The mutant PtoCPNαS260C/C290S was also used in this experiment. PtoCPNαS260C/C290S was immobilized on a gold‐coated substrate surface and labeled with a gold nanocrystal through the formation of a gold‐thiol bond. The twisting and tilting of PtoCPNα corresponded to Laue spots from the gold nanocrystals in the concentric circle (χ) and radial (θ) directions, respectively. Figure 8A shows the mean square angular displacement (MSD) in the presence of 0 mm and 1 mm ATP at each temperature. The MSD curve clearly showed the activation of PtoCPNα motion in tilting (θ) and twisting (χ). This may stem from the rotational motion of PtoCPNα, which is required for the folding function, and it is consistent with the results of ATP‐dependent GFP refolding activity and fluorescence change. Interestingly, both the θ and χ directional motion at 30 °C are more active than that at 50 °C. In particular, the motional difference in the θ direction is larger than that of the χ direction. The histogram of max angular displacement of θ direction clearly shows that the θ directional motion is enhanced at 30 °C (Fig. 8B). This result contradicts the idea that the θ directional motion is caused by Brownian motion. Thus, the open conformation of PtoCPNα is relatively unstable, and the fine motion of its apical domain is enhanced at lower temperatures, which might affect its interaction with the substrate.

### Temperature dependence of substrate release from PtoCPN

**Figure 9:**
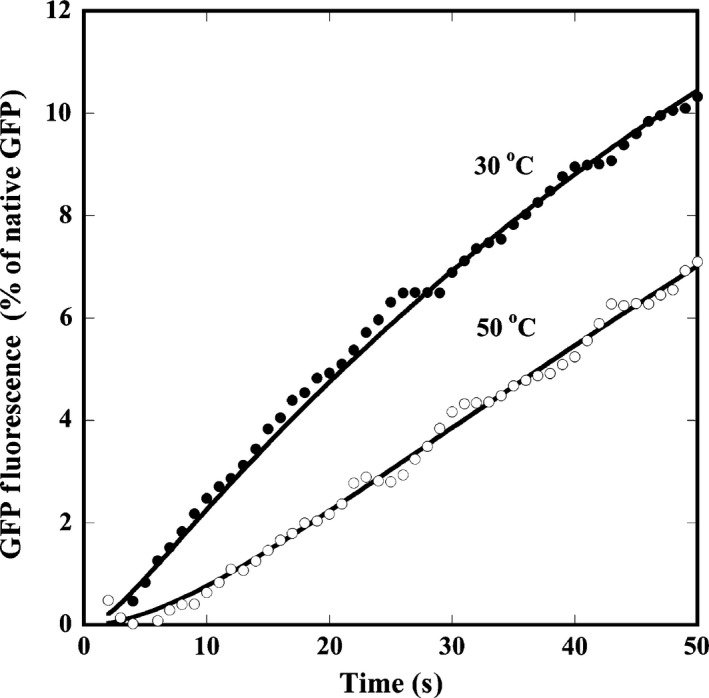
Release of GFP from the complex with PtoCPNα. Acid‐denatured GFP was diluted in the buffer containing PtoCPNα at 60 °C. The release and refolding of GFP was initiated by changing the temperature to 30 °C and 50 °C. The rate constants for the release of denatured GFP were calculated by fitting with the equation developed by the model. The details are described in the Materials and methods.

Finally, we examined the affinity of PtoCPN with denatured GFP by measuring the rate for the dissociation of its complex. The acid‐denatured GFP was diluted into the arresting buffer preincubated at 60 °C containing PtoCPNα. Because excess PtoCPNα was present in the buffer and the temperature was sufficiently high, the denatured GFPs were nearly completely captured by PtoCPN. After a 5‐min incubation, the mixture was diluted in assay buffer incubated at 30 °C or 50 °C. The temperature change promoted spontaneous GFP folding (Fig. 9). Compared with the results obtained at 30 °C, the GFP fluorescence gradually increased at 50 °C. The initial lag phase of GFP refolding likely reflected the release of denatured GFP molecules from PtoCPNα. Thus, we calculated the releasing rate constant assuming the following reaction model.

(Reaction 1) dGFP+PtoCPNα⇆dGFP−PtoCPNα

(Reaction 2) $$\text{dGFP}-\text{PtoCPN}\alpha \overset{k_{1}}{\underset{}{\to }}\text{dGFP}+\text{PtoCPN}\alpha$$

(Reaction 3) $$\text{dGFP}+\text{PtoCPN}\alpha \overset{k_{2}}{\underset{}{\to }}\text{GFP}+\text{PtoCPN}\alpha$$

First, PtoCPNα captures denatured GFP (dGFP) to form the dGFP‐PtoCPNα complex (dGFP‐PtoCPNα, Reaction 1). The dissociation of dGFP‐PtoCPNα complex occurs via a decrease in affinity at lower temperatures (Reaction 2). Next, the released dGFP is refolded spontaneously (Reaction 3). *k*_1_ and *k*_2_ are rate constants for the release of denatured GFP from PtoCPNα and the spontaneous folding of GFP, respectively. These processes are assumed to be irreversible here, and the rate constants were calculated by fitting to the data of the GFP fluorescence using the following equation:

$$A\left(t\right)=A\left(\infty \right)\times [1-\frac{1}{k_{2}-k_{1}}\times \left\{k_{2}e^{-k_{1}t}-k_{1}e^{-k_{2}t}\right\}]$$

where *A*(*t*) and *A*(∞) are the relative GFP fluorescence at time *t* and at the infinite time after the initiation of folding, respectively. The curve was well fitted by the described equation, and the calculated *k*_1_ values were 0.478 (± 0.118) at 30 °C and 0.118 (± 0.053) (S^−1^) at 50 °C. On the other hand, *k*_2_ values were calculated as 0.0196 (± 0.000862) and 0.0116 (± 0.00494) at 30 and 50 °C, respectively. These results suggested that the spontaneous folding of GFP should not be affected, but the affinity of PtoCPNα to substrate should be significantly weakened at lower temperatures.

## Discussion

*Picrophilus torridus* has two different genes of group II CPNs. On the basis of its homologies with group II CPNs of *Thermoplasma* spp. and *Thermococcus* spp., they are named as PtoCPNα and PtoCPNβ. The typical difference between α‐type and β‐type archaeal group II CPNs relates to the C‐terminal region. α‐type has Gly‐Gly‐Met repeats at the C terminus, which are nearly ubiquitously conserved in GroEL and archaeal group II CPNs. It is thought that the mildly hydrophobic Gly‐Gly‐Met repeat sequences protrude into the cavity from the bottom and function for the rapid folding of some proteins 30. However, the C‐terminal sequence of β‐type is relatively hydrophilic. PtoCPNs are highly homologous with those of *T. acidophilum*. It is known that the group II CPNs of *T. acidophilum* exist as hetero‐oligomers of α‐type and β‐type with a 1:1 stoichiometry. In contrast, *T*. KS1 CPN changes its stoichiometry. α‐type is dominant at its physiological temperature, and the content of β‐type increases with an increase in temperature. Thus, its homo‐oligomers represent the *T*. KS1 CPNs at moderate temperature and heat stressed conditions, respectively. Although it is not known whether homo‐oligomers of PtoCPNs are physiological oligomers, PtoCPNα, at least, exhibit characteristics of functional group II CPNs. As many arterial group II CPNs function as homo‐oligomers, the functional mechanism of PtoCPNα should reflect the natural group II CPNs. However, PtoCPNβ exists as a 14‐mer and exhibits marginal chaperone function. It might be possible that PtoCPNβ functions only as hetero‐oligomers complexed with PtoCPNα.

Although PtoCPNα exhibited high refolding activity for denatured GFP at its physiological temperature, 50 °C, it was unable to capture the denatured GFP at the relatively low temperature, 30 °C. However, PtoCPNα maintained the ability for ATP‐dependent conformational change at lowered temperatures. The fluorescence change indicated the ATP‐dependent conformation change in PtoCPNα from the open to the closed conformation at both 20 °C and 50 °C. The single‐molecule observation by DXT also suggested that the observed conformational change accompanied a twisting motion, which is required for folding function. Further analysis of DXT data showed that the tilting fine motion in the ATP‐free condition is enhanced at lower temperatures compared with those at the folding‐active temperature.

**Figure 10:**
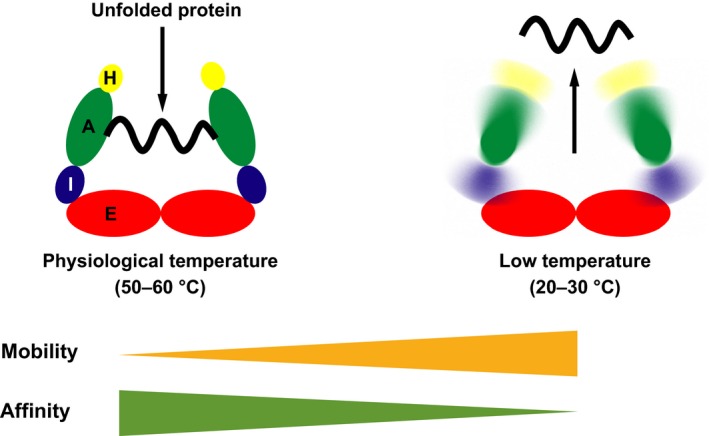
Schematic image for the effect of the conformational stability on the interaction with a denatured protein. Only one ring is shown. A, I, E, and H represent the apical domain, intermediate domain, equatorial domain, and the helical protrusion, respectively.

The affinity of group II CPNs for denatured proteins correlates with the mobility of its apical domain. Previously, our group attempted to generate *T*. KS1 CPN mutants in order to lower its functional temperature 9. Comparing the amino acid sequences of 26 thermophilic and 17 mesophilic CPNs, the amino acid residues that appeared to be related to their thermophilicity were selected and subjected to mutagenesis. Finally, *T*. KS1 CPN mutants with the amino acid replacements of E187D, K323R, and A523K were generated. In the GFP refolding assay, the *T*. KS1 CPN variants had the ability to capture the acid‐denatured GFP in ATP‐free conditions at 60 °C. Although wild‐type *T*. KS1 CPN lost the arresting ability of spontaneous refolding of acid‐denatured GFP at 40 °C, the *T*. KS1 CPN mutants retained the ability to arrest spontaneous GFP refolding. Importantly, the replaced amino acid residues were not located in the region where the denatured proteins bind. A523 is located at the β‐strand of the resolvable C‐terminal region, and E187 and K323 are located in the hinge region between the intermediate and apical domains. On the basis of this observation and an analysis of the crystal structure of TKS‐1 CPN, we concluded that these mutations changed the structural stability and mobility of TKS‐1 CPN and caused the adaptation of TKS‐1 CPN to relatively moderate temperatures. Taken together, these results clearly suggested that the conformational stability has an effect on the substrate‐binding abilities of group II CPNs (Fig. 10).

Here, we demonstrated that PtoCPNα increases their mobility at relatively low temperatures. These findings may correlate to the unfolding activity of chaperonins, which have not been previously reported 31.

## Author contributions

YY, YS, and MY conceived and designed the project. YY, KT, KN, NO, and HS acquired the data. YY, HS, YS, and MY analyzed and interpreted the data. YY and MY wrote the manuscript.
